# Effects of Dietary Mulberry Leaf Powder in Concentrate on the Rumen Fermentation and Ruminal Epithelium in Fattening Hu Sheep

**DOI:** 10.3390/ani9050218

**Published:** 2019-05-06

**Authors:** Jialiang Ouyang, Mengzhi Wang, Qirui Hou, Dan Feng, Yu Pi, Weiguo Zhao

**Affiliations:** 1 College of Animal Science and Technology, Yangzhou University, Yangzhou 225009, China; jialiangouyangyz@126.com; 2 The Seri-Cultural Research Institute, Chinese Academy of Agricultural Sciences, Zhenjiang 212018, China; houqr@163.com (Q.H.); zhaowgzj@163.com (W.Z.); 3 Department of Animal Science and Technology, Nanjing Agricultural University, Nanjing 210095, China; 2017105054@njau.edu.cn (D.F.); 2015205024@njau.edu.cn (Y.P.)

**Keywords:** mulberry leaf, rumen fermentation, rumen microbial, ruminal epithelial, Hu sheep

## Abstract

**Simple Summary:**

Rumen is the center of nutrient digestion, absorption and metabolism of ruminants. Ruminal microbes such as cellulolytic bacteria degrade the feed nutrients into smaller molecules, which are absorbed into the body mainly through rumen epithelium. Our study found that mulberry leaf powder supplementation improved the development of rumen epithelium, especially stratum basale, the most important layer of rumen epithelium for the energy metabolism. Our study suggests that supplementing ruminants with mulberry leaf powder may be one of the nutritional strategies that can improve the digestion and absorption performance of ruminants.

**Abstract:**

Mulberry leaves have been used as a protein source in replacing concentrates of domestic animals, however, little is known about the relationship between supplementation level and the development of rumen epithelium. This experiment aimed to investigate the effects of different proportions of mulberry leaf powder (MLP) in dietary concentrate on rumen fermentation and rumen epithelium morphology in fattening Hu sheep. Forty three-month-old male Hu sheep with an initial body weight of 16.5 ± 0.6 kg (BW ± SD) were chosen and randomly divided into five treatments: 0% (control), 15% (T15), 30% (T30), 45% (T45) and 60% (T60) of MLP in concentrate, respectively. The results showed that the dry matter intake (DMI) and average daily gain (ADG) in treatments T15 and T30 have no significant difference with respect to the control treatment, but DMI and ADG in treatments T45 and T60 were lower than the control treatment (*p* < 0.05). The apparent digestibility of organic matter (OM) and neutral detergent fiber (NDF) increased linearly and quadraticly as MLP supplementation increased (*p* < 0.05). The concentration of ammonia (NH_3_-N) trended to decrease linearly with the increase of MLP supplementation (*p* < 0.1), whereas the microbial protein (MCP) concentration increased linearly as MLP supplementation increased (*p* < 0.05). In the results of rumen epithelium morphology, the width of stratum corneum was reduced, whereas the width of ruminal papillae increased (*p* < 0.05), and the width of stratum granulosum and stratum basale also increased as MLP increased. In summary, MLP supplementation could improve nutrient digestibility, the development of rumen papillae and stratum basale. However, high content MLP (45%–60%) supplementation decreased the growth and food intake performance of fattening Hu sheep. Therefore, 30% MLP is recommended to supplement in concentrate for fattening Hu sheep.

## 1. Introduction

Mulberry leaves contain 82.7%–95.5% of carbohydrate, 24.6%–32.3% of neutral detergent fibers (NDF) and 15%–28% of crude protein (CP), similar to legume forages in chemical composition [1]. Mulberry leaves could be an exceptional forage for ruminants because of their high protein and nutritional value, as well as lower fiber and tannin contents [2]. Moreover, mulberry leaves have high total digestibility in vivo (in goats) and in vitro (80%–95%) [1], and even higher digestibility than alfalfa hay and oat hay in sheep [3]. On the other hand, the total tract apparent digestibility of starch from mixtures of 50% corn and 50% barley (on the DM basis) fed for lactating cows is 93.8% [4]. Therefore, mulberry leaves have been gradually used as a protein source in replacing concentrates in ruminants such as dairy cattle [1], beef cattle [5] and goat diets [6].

In ruminants, rumen bacterial species act as a more important factor in determining the extent and rate of feed degradation and utilization for the production of volatile fatty acid (VFA) in contrast to fungi and protozoa [7]. Tan et al. [8] reported that mulberry leaf meal along with urea can promote the population of ruminal cellulolytic bacteria of Brahman cattle. Niu et al. [9] also demonstrated that both mulberry leaves and mulberry fruit improve the abundance of total ruminal bacteria in finishing steers. Ruminal microbes degrade dietary carbohydrates to produce VFA, which acts as a chemical stimulating factor to promote the development of the rumen. If the VFA concentration is too low, the development of the rumen is inhibited as the lower VFA concentration could not meet the requirements for the growth of rumen papilla [10]. The morphologic traits on the rumen epithelium are the most important indicators of rumen development during the adaptation of rumen epithelium to VFA, as well as indicators of digestive, metabolic and absorptive capacity of rumen [11,12]. In turn, VFA affects the rumen morphology of animals especially the morphological structure of rumen epithelium [13]. Beiranvand et al. [14] reported that the morphological changes and branching of rumen papilla are mainly due to the stimulating effects of butyrate and propionate on the rumen cells. 

Although it has been demonstrated that mulberry leaves can be used as a protein source in replacing concentrates in ruminants, to date, there are no published studies reporting the effects of mulberry leaves on rumen morphological structure, which has been proven to play a central role in the nutrient absorption and metabolism of ruminants. Moreover, mulberry leaves exhibit a great amount of polyphenols which play key roles in the rumen morphology [12,15]. In view of these considerations, we hypothesize that mulberry leaf powder (MLP) supplementation can benefit and improve nutrient digestibility, ruminal fermentation and the development of rumen epithelium.

## 2. Materials and Methods

### 2.1. Animal and Diet

This project received ethical approval from the Animal Welfare Committee of Yangzhou Veterinarians of the Agriculture Ministry of China (Yangzhou, China, No. 201406018). Approximately three-month-old, 40 healthy fattening male Hu sheep were selected with similar genetic merit and body weight (BW ± SD) (16.5 ± 0.6 kg) from the Weihe Hu sheep farm in Siyang County (Jiangsu, China). Then they were randomly allocated into five treatments (n = 8 each) in a completely randomized design and fed with concentrate containing 0% (control), 15% (T15), 30% (T30), 45% (T45), and 60% (T60) MLP, respectively. In the first week, the concentrate was fed at 375 g/d, followed by an additional 25 g/d per week until the experiment finished. Whole plant corn silage was employed as a roughage source and provided at 1.5 kg/d during the first three weeks, then at 1.75 kg/d until the experiment ended. The diet was formulated to meet the nutrient requirements for a fattening sheep growing at 120 g/d (NRC, 2007). The concentrate ingredients and the nutrition contents of the diet are shown in Table 1.

Feed was offered twice daily (at 07:00 and 17:00) within individual pens. Water was available *ad libitum* through a papilla drinker in each pen. The feeding experiment was conducted in total 12 weeks, containing a 2 week adaptation period and a 10 week experimental period. The refusals of each sheep were collected and weighed every day, then feed intakes of individual sheep were calculated on a daily basis. The body weight (BW) of each sheep was recorded weekly in the early morning before feeding, and the average daily gain (ADG) was calculated as the regression of BW measured over time. The feed conversion ratio (FCR) was calculated as dry matter intake (DMI) divided by ADG. Three sheep were taken randomly from each treatment and slaughtered at the end of the experiment. These sheep were fasted for 24 h and were not given access to water for 2 h before transportation of about 30 min to a local slaughterhouse, where they were slaughtered in accordance with the Council Regulation (EC) No. 1099/2009 on the protection of animals at the time of killing.

### 2.2. Mulberry Leaf Powder Preparation

Mulberry (*Morus alba* var. *multicaulis* (Perrott.) Loud. China) leaves were collected in April of 2015 from a mulberry plantation in Zhenjiang, Jiangsu, China, then dried and ground according to Huyen, Wanapat and Navanukraw [5]. Briefly, mulberry leaves were collected and sun dried for about 3 days, then ground to pass a 1 mm screen using a Cyclotech Mill (Tecator, Höganäs, Sweden). The chemical composition of MLP was analyzed for dry matter (DM) (Association of Official Analytical Chemists (AOAC), 1990: method 934.01), ether extract (EE) (AOAC, 1990: method 920.39), ash (AOAC, 1990: method 942.05), calcium (Ca) (AOAC, 1990: method 985.35) and phosphorus (P) (AOAC, 1990: method 986.24). Crude protein (CP) content was determined using a Kjeldahl analyzer (Kjeltec 2300; FOSS Analytical AB, Hoganas, Sweden). NDF and acid detergent fiber (ADF) were measured using an Ankom Fiber Analyzer (Ankom Technology, Fairport, NY, USA) by following the procedure of Van Soest et al. [16]. The chemical composition (DM) of mulberry leaves was: DM, 89.54%; CP, 20.30%; EE, 8.15%; ash, 7.56%; NDF, 34.30%; ADF, 16.28%; Ca, 1.54% and P, 0.098%.

### 2.3. Sample Collection and Chemical Analysis

To determine the apparent total tract digestibility of nutrients, dietary samples and refusal samples were collected on a weekly basis. Fecal samples were collected randomly from six sheep of each treatment using fecal bags from day 31 to 35 and from day 61 to 65 of the experimental period. For each sheep and each sampling date, the collected fecal samples represent 15%–20% of total fresh feces. After drying at 65 °C to a constant mass in a forced air oven, the dietary, refusal and fecal samples were ground with a Retsch ZM 100 Wiley mill (Retsch GmbH, Haan, Germany) and passed through a 1 mm screen. The DM, CP, organic matter (OM), EE, NDF and ADF content of the dietary and fecal samples were analyzed using the same methods as mentioned above for determining these nutrient contents in mulberry leaves. Approximately 150 mL of rumen fluid sample was collected 2 h after morning feed from four sheep of each treatment by an esophageal tube vacuum pump sampling device (Anscitech Company, Wuhan, China) at the 14, 28, 42, 56 and 70 day marks of the experimental period. Collected rumen fluid was filtered through four layers of cheesecloth to immediately measure the pH value using a pHS-3C electrode pH meter (Shanghai Chemical Company, Shanghai, China), then stored at −20 °C until the determinations were found of the concentrations of ammonia nitrogen (NH_3_-N) using the method of Rhine et al. [17], microbial crude protein (MCP) according to Wang et al. [18], total VFA (TVFA) and individual VFA. The TVFA and individual VFA concentration was analyzed by a gas chromatography (GC-17 A, Shimadzu, Tokyo, Japan) according to the method of Wang et al. [19]. Briefly, 1 mL of 25% metaphosphoric acid and 1 mL of 0.6% 2-ethylbutyric acid (internal standard) were added into 5 mL rumen fluid samples and mixed properly. The mixed samples were centrifuged three times at 4000× *g* for 30 min at 4 °C to take 1 μL clear supernatant and injected into a Supelco Nukol silica capillary column (length 30 m, inner diameter 0.53 mm, film thickness 0.5 μm). The temperature of the injector was set at 220 °C and the detector temperature was set at 250 °C. The temperature programing of the column was heated from 60 °C to 190 °C at a speed of 20 °C·min^−1^. After slaughter at the end of the experiment, a 1 × 1 cm section of the rumen wall was cut from each sheep, then the samples were rinsed with phosphate buffer saline and immediately placed in a 4% neutral formalin for morphometric analysis.

### 2.4. Morphometric Analysis

The rumen morphology was observed by paraffin section and hematoxylin-eosin staining (HE). Tissues were prepared according to the method described by Wang et al. [20]. Briefly, formalin-fixed tissues were dehydrated in ascending concentration of absolute ethanol (50%, 70%, 80%, 90%, 100%), cleared with xylene twice, then infiltrated with and embedded in paraffin. 7 μm-thick tissue slices (10 slices of each sample) were generated by a rotary microtome then stained with HE. The morphometric structure of the sections was observed by an optical microscope (Olympus, Tokyo, Japan). The width of the rumen cell layer (stratum corneum (SC), stratum granulosum (SG), stratum spinosum (SS) and stratum basale (SB)) was measured at a magnification of 20×, and the width of the rumen papilla was calculated at a magnification of 4× using the Olympus IX71 microscope software cellSens Dimensio (version 1.0, Olympus, Tokyo, Japan). Both of the width of rumen cell layer and papilla were measured at six randomly chosen sites within each slide.

### 2.5. Statistical Analysis

Data for body growth, DMI, digestibility and rumen fermentation parameters were analyzed by using PROC MIXED of SAS 9.2 (SAS Institute Inc, Cary, NC, USA) for repeated measures according to the statistical model:

Y_ijk_ = μ + M_i_ + T_j_ + S_k_ + MT_ij_ + ε_ijk_

where Y_ijk_ is the dependent variable, μ is the overall mean, M_i_ is the fixed effect of MLP treatment, T_j_ is the fixed effect of time, S_k_ is the random effect of sheep, MT_ij_ is the fixed interaction effect between MLP treatment and time and ε_ijk_ is the random error term. Data for morphometric parameters of rumen were analyzed by PROC GLM of SAS 9.2. Tukey’s multiple range test was applied to MLP treatment means which showed a statistically significant variation in the samples, and polynomial orthogonal contrasts were applied to linear, quadratic effects of MLP treatment. In all cases, the individual sheep was considered as the experimental unit. Significance was declared at *p* ≤ 0.05 and tendency at 0.05 < *p* < 0.1.

## 3. Results

### 3.1. Dry Matter Intake (DMI) and Average Daily Gain (ADG) of Fattening Hu Sheep Fed with Mulberry Leaf Powder

As shown in the Table 2, the final BW, DMI and ADG in treatments T45 and T60 were significantly lower (*p* < 0.05) than those in the control treatment and the other two treatments (T15 and T30). The final BW and ADG decreased linearly, while feed conversion ratio (FCR) increased as MLP supplementation increased in dietary concentrate (*p* < 0.05). However, DMI and ADG showed no significant difference in treatments T15 and T30 as compared with the control treatment.

### 3.2. Apparent Digestibility of Nutrients of Fattening Hu Sheep Fed with Mulberry Leaf Powder

As shown in the Table 3, total tract apparent digestibility of DM, CP, ADF and EE showed no significant differences among the treatments, but the apparent digestibility of OM in treatments T15, T30 and T45 was higher (*p* < 0.05) than the control treatment. Apparent digestibility of NDF in treatments T30, T45 and T60 was higher (*p* < 0.05) than that in the T15 treatment. However, apparent digestibility of OM and NDF increased linearly and quadratically with the increase of MLP supplementation (*p* < 0.05). 

### 3.3. Rumen Fermentation Parameters of Fattening Hu Sheep Fed with Mulberry Leaf Powder

The differences in rumen pH value and MCP concentration were not significant among experimental treatments (Table 4). The NH_3_-N concentration trended to decrease linearly with the increase in MLP supplementation (*p* < 0.1), in contrast, MCP concentration increased linearly (*p* < 0.05). TVFA and acetate concentrations trended to increase linearly as MLP supplementation was increased (*p* < 0.1). The ratio of acetate concentration to propionate concentration linearly increased whereas the propionate concentration linearly decreased (*p* < 0.05).

### 3.4. Morphological Variables of Rumen Epithelial Tissue of Fattening Hu Sheep Fed with Mulberry Leaf Powder

**Figure 1:**
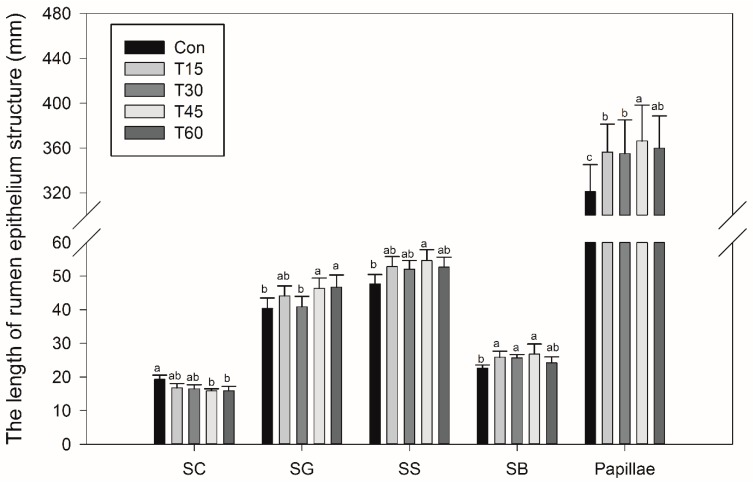
Morphometric variables of rumen epithelium structure in fattening Hu sheep fed with mulberry leaf powder. SC = Stratum corneum; SG = Stratum granulosum; SS = Stratum spinosum; SB = Stratum basale. ^a–c^ Bar chart within same epithelium structure with different superscripts differ significantly at *p* ≤ 0.05.

**Figure 2:**
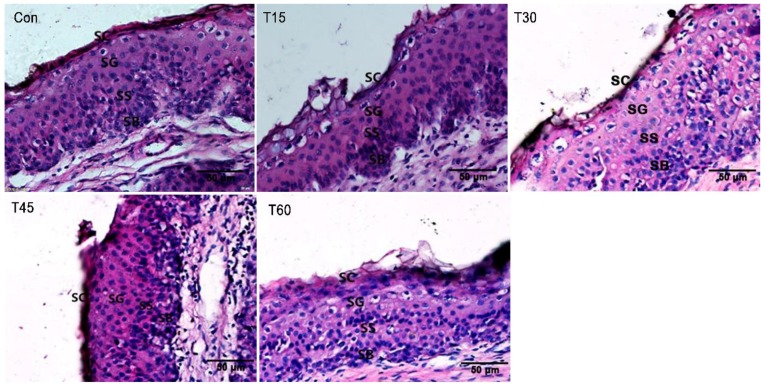
Morphological structure of rumen epithelium in fattening Hu sheep fed with mulberry leaf powder (20×). SC = Stratum corneum; SG = Stratum granulosum; SS = Stratum spinosum; SB = Stratum basale.

**Figure 3:**
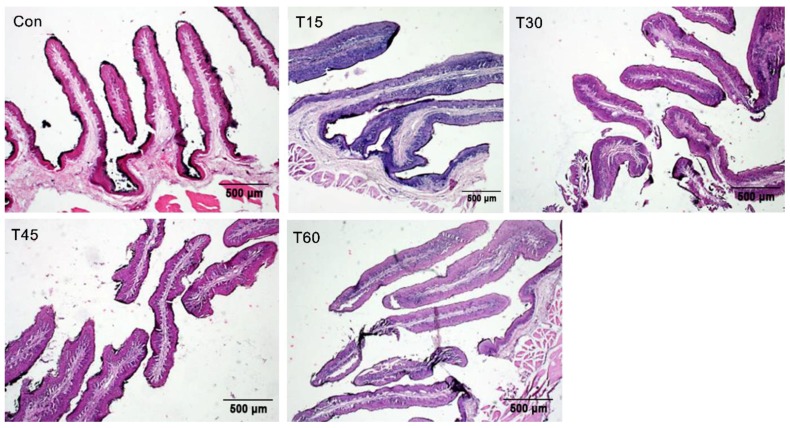
Morphological structure of rumen papillae in fattening Hu sheep fed with mulberry leaf powder (4×).

The width of the rumen papilla, SC, SG, SS and SB is shown in the Figure 1. The width of SC decreased (*p* = 0.04), whereas the width of ruminal papillae increased linearly as MLP supplementation increased (*p* = 0.03). The width of SG (*p* = 0.06) and SB (*p* = 0.08) also trended to increase. Moreover, the width of SS in T45 treatment was higher than the control treatment (*p* < 0.05). The morphological structure of rumen epithelium (20×) is shown in Figure 2 and the morphological structure of rumen papillae (4×) is shown in Figure 3.

## 4. Discussion

Mulberry leaves not only contain high protein content, but also high palatability [1]. Anbarasu, Dutta, Sharma and Rawat [6] demonstrated that leaf meal within mulberry leaves can attain the same DMI and ADG as groundnut cake fed to goats. In the current study, the DMI and ADG of 15%–30% MLP treatments showed no significant difference when compared with the control treatment, which shows that MLP (15%–30%) can partially be used as concentrate for Hu sheep. However, we also found that high contents of MLP supplementation ranging from 45% to 60% decreased the DMI and ADG. These results are consistent with the findings of lactating Holstein cows, where it was shown that the milk yield and food intake decreased with the increased content of mulberry meal in the concentrate [1]. We make the explanation that a high content of MLP supplementation increases the content of dietary fiber in concentrate, which may decrease the diet palatability, so as to decrease the DMI and ADG even the milk yield [21].

Mulberry leaves are nutritious, with 89.54% of dry matter and 20.30% of crude protein, as this was determined on a DM basis. The 7.56% of ash (on a DM basis) indicates that mulberry leaves contain a large amount of OM. Dietary carbohydrates are the most important organic nutrients for ruminants, as the rumen microorganisms use them to produce a large amount of VFA. Fiber is also very important in maintaining the normal rumen environment, as it is slowly digested solid fraction [16]. It has been shown that NDF intake is positively related to the daily rumination and body size of ruminants, as it is the only solution to recover insoluble matrix carbohydrate [16]. Studies also show that a mulberry leaf diet helps to promote the apparent digestibility of NDF in beef cattle and sheep [5,22]. Kramer-Schmid et al. [23] found that the digestibility of OM, DM, body weight and milk yield increased along with the enhanced NDF digestibility in whole crop maize silage. However, Huyen, Wanapat and Navanukraw [5] reported that 400–600 g/head/day supplementation of mulberry leaf pellet with a rice straw-based diet helps to promote the OM, NDF and ADF digestibility in beef cattle. Chen et al. [24] demonstrated that feeding sheep with a mulberry leaf flavonoid diet can significantly promote the apparent digestibility of OM and NDF, while reducing methane emission. Our study in Hu sheep is also in consistent with these findings. However, the apparent digestibility of DM, CP, ADF and EE showed no significant differences among four MLP treatments with respect to the control diet, indicating that it is practicable to use MLP by replacing partial contents of concentrate.

Concentrate is the main resource of nutrient and energy supply to animals, but ruminal lactate-producing bacteria could ferment the starch to lactate when ruminants were fed too much high grain concentrate to meet the energy requirement. The accumulation of lactic acid could reduce the pH value of rumen fluid and lead to ruminal acidosis, which in turn damages the animal’s health and depresses production performance [25]. MLP contains low starch but large quantities of fiber, and the content of fiber, especially physically effective fiber, in feed plays a very important role in preventing ruminal acidosis [26]. Therefore, we speculated MLP may could be used to prevent the ruminal acidosis. Moreover, flavonoid, as an abundant bioactive compound in mulberry leaf, could also reduce the risk of ruminal acidosis through increasing the populations of lactate-consuming bacteria *Megasphaera elsdenii* in the rumen fluid of young ruminants [27,28]. In this study, the rumen pH value was shown to not be affected by MLP supplementation, which is in accordance with the optimum range of 6.3–7.4 for rumen microorganisms and environmental factors [29]. 

The results on NH_3_-N and MCP concentration in the current study showed that MLP supplementation may promote the nitrogen efficiency, as a large amount of NH_3_-N was transformed to MCP [30]. Both the acetate production and the ratio of acetate concentration to propionate concentration increased due to the higher content of fiber in the diet, leading to the decreased efficiency of metabolic energy utilization for fattening [31]. However, as the ratio of acetate concentration to propionate concentration in 15%–30% MLP treatments was higher than the control treatment, our study showed that these treatments had no influence on the DMI and ADG of Hu sheep. This was because the high content protein in MLP could supply enough metabolic energy to the animals [1]. However, ruminal microorganisms not only play the most important role in the synthesis of MCP, but also in transforming carbohydrates to VFA through ruminal anaerobic fermentation. Tan, Wanapat, Uriyapongson, Cherdthong and Pilajun [8] reported that a mulberry leaf diet can stimulate the growth and multiplication of ruminal microorganisms. Therefore, we speculated that MLP could improve the nitrogen efficiency and ruminal VFA metabolism of ruminants through influencing the community structure or abundances of ruminal microorganisms.

Acetate and butyrate in the rumen are not only the precursor substances of fat synthesis especially for the milk fat in dairy animals, but also play a critical role in stimulating the development of rumen epithelium [32]. Rumen epithelium consisting of SC, SG, SS and SB possess several vital physiological functions, including VFA metabolism, absorption, transportation and protection [33,34]. Ragionieri et al. [35] reported that high proportions of acetate in rumen can promote the rapid development of rumen epithelium and decrease the width of SC. In this experiment, the width of SC decreased, whereas the width of ruminal papillae, SG and SB increased. As for the rumen epithelium structure, SB seems to be the most important ruminal layer for energy metabolism, as the basale cells contribute to metabolic properties such as ketogenesis [36]. Moreover, the cells of SG may establish tight gap junctions that maintain the integrity of metabolite concentration gradients across the rumen wall [32]. Therefore, these results indicate that MLP supplementation might stimulate the development of SB and SG as well as the metabolism capacity of rumen epithelium. All of these results are consistent with the findings of growing lambs fed dietary *Urtica cannabina*, which was found to improve the rumen epithelium through increasing acetate production in rumen [12]. Both *Urtica cannabina* and MLP not only contain high content of dietary carbohydrate, which could be fermented into ruminal acetate, but also a high content of polyphenols that play an important role in improving the development of rumen epithelium [12,15].

## 5. Conclusions

In conclusion, MLP supplementation can promote nutrient digestibility in the rumen. This supplementation can also enhance the acetate concentration in rumen fermentation, and as a result can promote the development and the metabolic properties of rumen epithelium. These results suggest that it is practicable to employ MLP to partially replace dietary concentrate. However, the high content MLP (45%–60%) supplementation decreased growth and food intake performance of fattening Hu sheep. Therefore, 30% MLP is recommended to supplement the concentrate for fattening Hu sheep.

## Figures and Tables

**Table 1:** Concentrate composition and nutrition content of diets to fattening Hu sheep (on the DM basis) %.

Items	Control	T15	T30	T45	T60
Concentrate ingredient					
Mulberry leaf powder		15.0	30.0	45.0	60.0
Corn	30.0	30.5	33.5	21.0	7.3
Soybean meal	27.0	25.5	28.0	24.0	15.5
Wheat bran	16.3	9.8	2.5		
Brown rice	14.2	9.0			
*Saccharomyces cerevisiae* ^1^	6.7	3.5		1.5	5.0
Corn protein meal	2.0	3.0	1.5		
CaH_2_PO_4_	1.2	1.2	1.2	1.0	1.5
Fatty powder			1.0	5.3	8.7
Limestone	1.6	1.5	1.3	1.2	1.0
Salt	0.5	0.5	0.5	0.5	0.5
Premix ^2^	0.5	0.5	0.5	0.5	0.5
Total	100.0	100.0	100.0	100.0	100.0
Nutrition content (g/kg)					
Apparent DE (MJ/kg) ^3^	14.3	13.8	13.9	13.9	13.5
Crude protein	251.1	249.8	249.8	251.3	250.4
Neutral detergent fiber	159.7	159.9	155.7	155.1	164.5
Calcium	9.6	9.9	10.0	9.9	10.6
Phosphorus	6.8	6.4	6.1	5.6	6.8

^1^*Saccharomyces cerevisiae*: A species of yeast culture. ^2^ Composition of premix (mg/kg): P, 5; Se, 5; CuSO_4_, 200; FeSO_4_▪H_2_O, 1500; ZnSO_4_▪H_2_O 1000; CoCl_2_ 5; Vitamin A, 200,000 IU/kg; Vitamin D, 1 250,000 IU/kg. ^3^ Apparent digestible energy (DE) that was calculated by the difference between dietary gross energy and fecal gross energy.

**Table 2:** Dry matter intake (DMI) and average daily gain (ADG) of fattening Hu sheep fed with mulberry leaf powder.

Item	Treatments					SEM	*p*-Value	
	Control	T15	T30	T45	T60		Linear	Quadratic
Initial BW (kg)	17.13	16.97	16.98	16.91	17.04	0.426	0.72	0.98
Final BW (kg)	23.88 ^a^	23.69 ^a^	23.37 ^a^	22.51 ^b^	21.91 ^c^	0.528	0.02	0.09
ADG (kg/d)	0.12 ^a^	0.12 ^a^	0.11 ^a^	0.10 ^b^	0.09 ^c^	0.005	0.02	0.20
DMI (kg/d)	0.87 ^a^	0.86 ^a^	0.86 ^a^	0.80 ^b^	0.82 ^b^	0.014	0.07	0.91
FCR	7.23 ^c^	7.15 ^c^	7.54 ^c^	8.01 ^b^	9.39 ^a^	0.137	0.03	0.05

BW = body weight; ADG = average daily gain; DMI = dry matter intake; FCR = feed conversion ratio. ^a–c^ Values within a row with different superscripts differ significantly at *p* ≤ 0.05.

**Table 3:** Apparent digestibility of nutrients of fattening Hu sheep fed with mulberry leaf powder %.

Item	Treatments					SEM	*p*-Value	
	Control	T15	T30	T45	T60		Linear	Quadratic
DM	61.46	63.17	66.89	67.12	64.00	1.833	0.10	0.17
OM	50.52 ^b^	50.06 ^b^	60.86 ^a^	63.23 ^a^	65.54 ^a^	3.120	0.03	<0.01
CP	72.01	72.63	75.03	76.21	73.93	1.872	0.18	0.38
ADF	47.35	48.38	48.89	52.00	53.79	3.781	0.95	0.13
NDF	50.87 ^b^	54.22 ^ab^	59.13 ^a^	61.21 ^a^	66.73 ^a^	2.549	0.02	0.04
EE	76.06	73.16	82.46	77.68	85.91	4.414	0.74	0.16

DM = dry matter; OM = organic matter; CP = crude protein; ADF = acid detergent fiber; NDF = neutral detergent fiber; EE = ether extract. ^a,b^ Values within a row with different superscripts differ significantly at *p* ≤ 0.05.

**Table 4:** Rumen fermentation parameters in rumen fluid of fattening Hu sheep fed with mulberry leaf powder.

Item	Treatments					SEM	*p*-Value	
	Control	T15	T30	T45	T60		Linear	Quadratic
pH	6.71	6.70	6.80	6.78	6.88	0.067	0.54	0.31
NH_3_-N (mg/dL)	18.28 ^a^	18.99 ^a^	16.85 ^a^	12.24 ^b^	10.65 ^b^	1.023	0.08	0.16
Microbial protein (MCP) (mg/mL)	0.62	0.66	0.70	0.72	0.79	0.060	0.04	0.25
TVFA ^1^ (mM)	86.72 ^ab^	93.10 ^a^	91.74 ^a^	84.29 ^ab^	79.08 ^b^	3.263	0.44	0.77
Acetate (mol/100 mol)	69.84 ^b^	69.98 ^b^	70.91 ^ab^	72.56 ^a^	71.90 ^a^	0.611	0.08	0.30
Propionate (mol/100 mol)	17.35 ^a^	17.28 ^a^	16.13 ^ab^	16.20 ^ab^	15.98 ^b^	0.350	<0.01	0.77
Butyrate (mol/100 mol)	9.86 ^ab^	10.07 ^a^	9.98 ^ab^	8.68 ^b^	9.15 ^b^	0.436	0.93	0.10
Acetate:Propionate	4.02 ^b^	4.05 ^b^	4.41 ^ab^	4.51 ^a^	4.49 ^a^	0.109	<0.01	0.53

^1^ TVFA (total volatile fatty acids) = acetate + propionate + butyrate + valerate + isobutyrate + isovalerate. ^a,b^ Values within a row with different superscripts differ significantly at *p* ≤ 0.05.
